# 

**Published:** 1876-11

**Authors:** 


VOL. VII. NOVEMBER, 1876. No. 11.

### THE SOUTHERN
### MEDICAL RECORD

PUBLISHED MONTHLY AT

ATLANTA, GEORGIA.

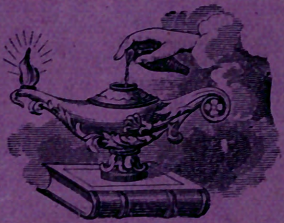

LOCAL EDITORS:

THOS. S. POWELL, M.D. R. C. WORD, M.D

W. T. GOLDSMITH, M.D. H. B. LEE, M.D.

ASSOCIATE EDITORS:

T. URTIS SMITH, M.D. .........MIDDLEPORT, OHIO.
SWAN M. BURNETT, M.D .......KNOXVILLE, TENN.
ALBAN S. PAYNE, M.D...........MARKHAM'S, VA.
A. R. KILPATRICK, M.D.........NAVASOTA, TEXAS.
A. A. LYON, M.D.... ............SHREVEPORT, LA.
G. A. BAXTER, M.D ..............CHATTANOOGA, TENN.
J. B. MATTISON, M.D............BROOKLYN, N. Y.
E. T. EASLEY, A.M., M.D..... ...LITTLE ROCK, ARK.

*"QUICQUID PRÆCIPIES ESTO BREVIS."*

ATLANTA, GA.:

JAS. P. HARRISON & CO., PRINTERS AND BINDERS.

1876.

